# Kidney Disease Associated With Mono-allelic *COL4A3* and *COL4A4* Variants: A Case Series of 17 Families

**DOI:** 10.1016/j.xkme.2023.100607

**Published:** 2023-02-01

**Authors:** Sander Groen in ’t Woud, Ilse M. Rood, Eric Steenbergen, Brigith Willemsen, Henry B. Dijkman, Michel van Geel, Jeroen Schoots, Jack F.M. Wetzels, Dorien Lugtenberg, Jeroen K.J. Deegens, Ernie M.H.F. Bongers

**Affiliations:** 1 Department of Human Genetics, Radboud University Medical Center, Nijmegen, The Netherlands; 2 Department for Health Evidence, Radboud University Medical Center, Nijmegen, The Netherlands; 3 Department of Nephrology, Radboud University Medical Center, Nijmegen, The Netherlands; 4 Department of Pathology, Radboud University Medical Center, Nijmegen, The Netherlands; 5 Department of Clinical Genetics, Maastricht University Medical Center, Maastricht, The Netherlands

**Keywords:** Alport syndrome, *COL4A3/COL4A4*, FSGS, genotype-phenotype, mono-allelic variants, type IV collagen nephropathy, whole exome sequencing

## Abstract

**Rationale & Objective:**

Mono-allelic variants in *COL4A3* and *COL4A4* (*COL4A3/COL4A4*) have been identified in a spectrum of glomerular basement membrane nephropathies, including thin basement membrane nephropathy and autosomal dominant Alport syndrome. With the increasing use of next generation sequencing, mono-allelic *COL4A3/COL4A4* variants are detected more frequently, but phenotypic heterogeneity impedes counseling. We aimed to investigate the phenotypic spectrum, kidney biopsy results, and segregation patterns of patients with mono-allelic *COL4A3*/*COL4A4* variants identified by whole exome sequencing.

**Study Design:**

Case series.

**Setting & Participants:**

We evaluated clinical and pathologic characteristics of 17 Dutch index patients with mono-allelic variants in *COL4A3*/*COL4A4* detected by diagnostic whole exome sequencing and 25 affected family members with variants confirmed by Sanger sequencing.

**Results:**

Eight different mono-allelic *COL4A3/COL4A4* variants were identified across members of 11 families, comprising 7 glycine substituted missense variants and 1 frameshift variant. All index patients had microscopic hematuria at clinical presentation (median age 43 years) and 14 had (micro)albuminuria/proteinuria. All family members showed co-segregation of the variant with at least hematuria. At end of follow-up of all 42 individuals (median age 54 years), 16/42 patients had kidney function impairment, of whom 6 had kidney failure. Reports of kidney biopsies of 14 patients described thin basement membrane nephropathy, focal segmental glomerulosclerosis, minimal change lesions, and Alport syndrome. Electron microscopy images of 7 patients showed a significantly thinner glomerular basement membrane compared with images of patients with idiopathic focal segmental glomerulosclerosis and other hereditary glomerular diseases. No genotype-phenotype correlations could be established.

**Limitations:**

Retrospective design, ascertainment bias toward severe kidney phenotypes, and familial hematuria.

**Conclusions:**

This study confirms the wide phenotypic spectrum associated with mono-allelic *COL4A3/COL4A4* variants, extending from isolated microscopic hematuria to kidney failure with high intra- and interfamilial variability.

**Plain Language Summary**

Variants in the *COL4A3* and *COL4A4* genes are found more frequently nowadays because of increased use of genetic testing. In this study of 42 individuals with a variant in one of the alleles of the *COL4A3/COL4A4* gene from 17 Dutch families, we investigated kidney disease symptoms and kidney biopsies. The most important findings were that the kidney disease symptoms were highly variable, ranging from mild (only hematuria) to very severe (kidney failure necessitating kidney replacement therapy), both within and between families. In addition, the glomerular basement membrane was found to be thinner in patients with a variant in *COL4A3/COL4A4* than in patients with other kidney disorders. No correlation between disease severity and the results of genetic testing could be established.

Variants in the *COL4A3* and *COL4A4* genes cause aberrant collagen IV synthesis resulting in ultrastructural changes of the glomerular basement membrane (GBM). This underlies a wide spectrum of inherited kidney diseases varying from thin basement membrane nephropathy to Alport syndrome. Bi-allelic variants in *COL4A3* and *COL4A4* cause autosomal recessive Alport syndrome, characterized by progressive kidney failure, sensorineural hearing loss, and ocular abnormalities. In contrast, mono-allelic variants in *COL4A3* and *COL4A4* have been described as resulting in benign familial hematuria or thin basement membrane nephropathy,^1^^,^^2^ but have also been identified in patients with characteristic Alport lesions on kidney biopsy who progressed to kidney failure, which was subsequently designated as autosomal dominant Alport syndrome.^3^, ^4^, ^5^, ^6^ Delineation of the clinical spectrum and the phenotypic heterogeneity in individuals with mono-allelic *COL4A3* and *COL4A4* variants is essential for counseling patients and at-risk family members regarding follow-up, expected prognosis, therapy, and the inheritance pattern of their disease.^7^ It will also contribute to developing and establishing uniform and univocal nomenclature of kidney diseases associated with these mono-allelic variants in the diagnostic workup.^8^^,^^9^

In the past, diagnostic testing was limited to kidney biopsy and single gene testing. In focal segmental glomerulosclerosis (FSGS) patients, *COL4A3* and *COL4A4* were frequently not tested, resulting in a missed molecular diagnosis in some patients and inappropriate genetic counseling.^10^^,^^11^ In the current era of next generation sequencing, a mono-allelic variant in *COL4A3* or *COL4A4* is found frequently in patients diagnosed with FSGS.^12^, ^13^, ^14^ Recent findings from the 100,000 Genomes Project even indicate that mono-allelic *COL4A3* and *COL4A4* variants are present in ∼1% of Europeans, which underlines the importance of clinical studies of individuals with these variants.^15^

Although many different variants in *COL4A3* and *COL4A4* have been identified, the genetic pathogenicity and phenotypic heterogeneity is still unresolved, and *COL4A3* and *COL4A4* variants of unknown significance challenge genetic counseling. High intra- and interfamilial variability of kidney function and disease severity has been observed in families in which mono-allelic *COL4A3* or *COL4A4* variants co-segregate with kidney disease, suggesting that other factors like genetic modifier variants, epigenetic modulation, and/or environmental factors may modify the severity of disease.^10^^,^^16^, ^17^, ^18^, ^19^, ^20^, ^21^, ^22^ In this study, we performed an in-depth analysis of the kidney phenotype, kidney biopsy results and exome data in a cohort of patients and affected family members with a mono-allelic *COL4A3* or *COL4A4* variant. The aim of this study was three-fold: (1) to delineate the clinical spectrum of mono-allelic variants in *COL4A3* or *COL4A4*; (2) to identify genotype-phenotype correlations for the identified variants; and (3) to characterize kidney histology of patients with a mono-allelic variant in *COL4A3* and *COL4A4* and compare this with other causes of FSGS and other inherited kidney diseases.

## Methods

### Patients

Diagnostic genetic testing at the genome diagnostics laboratory of the Radboud university medical center was performed in 72 patients with a suspected hereditary glomerular disease based on the presence of 1 or more of the following clinical features: persistent or recurrent glomerular hematuria, proteinuria, (steroid resistant) nephrotic syndrome, and a positive family history of kidney disease. In 17 of 72 unrelated Dutch patients (24%), a mono-allelic *COL4A3* or *COL4A4* variant was identified using whole exome sequencing, and these patients were included in this study as index patients. One index patient and his family (family 11) had been tested and published previously with benign hematuria.^1^ Patients were referred to the Radboudumc Center of Expertise for Rare Kidney Diseases between June 2013 and December 2016. Two patients with bi-allelic *COL4A3* or *COL4A4* variants and their family members were excluded from analyses.

Clinical, pathological, and molecular diagnoses of the index patients were retrieved from medical records and last updated in December 2021. For patients who were not under follow-up at our center, medical information was requested from their nephrologist using a questionnaire. All index patients gave written or oral consent for analysis of the kidney disorder gene panel by whole exome sequencing after counseling by a clinical geneticist or nephrologist. In case of simultaneous analysis of the kidney disorder gene panel and the entire exome, the index patients received pre- and post-test counseling by a clinical geneticist, and written informed consent was obtained. The Medical Review Ethics committee Arnhem-Nijmegen approved whole exome sequencing under the realm of clinical diagnostic genetic testing (2011/188).

### Whole Exome Sequencing

DNA was extracted from peripheral blood samples and enriched with the Agilent SureSelectXT Human All Exon 50Mb Kit. Whole exome sequencing was performed using an Illumina HiSeq2000TM or HiSeq4000TM machine at BGI-Europe in Copenhagen, Denmark. Following read alignment with BWA and variant calling with GATK, variants were annotated using an in-house developed program at the department of Genetics of the Radboud university medical center. At first, only genes in the kidney disorders gene panel were selected (up to 250 genes at time of genetic testing).^23^ This panel contains genes for which evidence of a relation with kidney disorders is available in the literature. Data on the frequency of variants in control populations (<5% in dbSNP and <1% in an in-house database), nucleotide and amino acid conservation, inheritance pattern, and the phenotype associated with the genes were combined to prioritize variants. All reported variants that did not meet our validated quality standard were confirmed using Sanger sequencing. The genes *COL4A3* and *COL4A4* were covered >20× for 95% (>10× for 98%). The pathogenicity of detected DNA variants was assessed using the American College of Medical Genetics and Genomics 2015 guidelines for clinical sequence interpretation.^24^ A systematic copy number variation analysis was performed using copy number variation calling software (CoNIFER) to detect deletions and duplications of at least 3 consecutive exons.^25^ Exome-wide analysis was performed in 5 index patients to exclude other causative variants explaining the kidney phenotype. No additional variants were found in these patients.

### Evaluation of Pathogenicity and Segregation Analysis by Sanger Sequencing

As part of the diagnostic workup, segregation analysis of variants in *COL4A3* (NM_000091.4) or *COL4A4* (NM_000092.4) was performed by Sanger sequencing for 2 different purposes. First, after the identification of known (likely) pathogenic variants for autosomal recessive Alport syndrome, segregation analysis was performed to gain more insight into the role of a mono-allelic variant within a family with hematuria, proteinuria, and in some cases, kidney failure. Secondly, in the case of novel unpublished variants, segregation analysis was also performed to evaluate the pathogenicity of the variant by testing co-segregation of the variant and the kidney phenotype. Medical information from first and second degree family members was requested after written informed consent was obtained from the concerning family member and/or their legal caregivers. Family members were added to our study cohort if the familial *COL4A3* or *COL4A4* variant was detected by segregation analysis.

For Sanger sequencing, genomic DNA was extracted from leukocytes from ethylenediaminetetraacetic acid blood samples and purified using standard DNA extraction methods. Amplicons harboring the variants of interest were amplified using exon-specific polymerase chain reaction primers (primer sequences are available upon request). Polymerase chain reaction was performed using Amplitaq Gold DNA polymerase on a GeneAmp PCR 9700 system. After purification using Millipore plates, Sanger sequencing was performed using an ABI3730XL platform (Thermo Fisher Scientific).

### Evaluation of Pathology Reports of Kidney Biopsies

If available, pathology reports of kidney biopsies were reviewed and, if available, kidney specimens were re-examined by a kidney pathologist. Electron microscopy (EM) images of kidney biopsies of patients with a mono-allelic variant in *COL4A3*/*COL4A4* were blindly compared with images from adult patients with other glomerular diseases (i.e., hereditary nephropathy caused by a variant in a gene other than *COL4A3/COL4A4*, steroid resistant nephrotic syndrome without a genetic diagnosis (kidney gene panel by whole exome sequencing negative for a disease-causing DNA variants or steroid responsive idiopathic FSGS without a genetic test). The images were evaluated for GBM structure, thickness (measured at approximately 5 points per image in all available EM images), and consistency of thickness (defined as consistent, low variability, variable, or high variability). Furthermore, the extent of podocyte effacement (none, ≤80%, or >80%) and endothelial injury (none, moderate, or severe) was evaluated.

### Statistical Analysis

Values are given as median with range. Differences between patient groups were analyzed using Mann-Whitney *U* tests for continuous variables and Fisher exact test for categorical variables. *P* values below 0.05 were considered statistically significant.

## Results

### Clinical Characteristics

Mono-allelic variants in *COL4A3* were identified in 5 index patients and in *COL4A4* in 12 index patients (Table 1).^26^, ^27^, ^28^, ^29^, ^30^, ^31^ All index patients had a positive family history of kidney disease. The median age at clinical presentation in index patients was 43 years (range 4-55; Table 2). The median estimated glomerular filtration rate (eGFR) at presentation, calculated using the Chronic Kidney Disease Epidemiology Collaboration (CKD-EPI) formula, was 103 mL/min/1.73 m^2^ (range 21-110), including 4 patients with an eGFR below 60 mL/min/1.73 m^2^. All index patients had microscopic hematuria, and 14 of 17 patients (82%) had (micro)albuminuria/proteinuria at clinical presentation. Nephrotic range proteinuria (defined as ≥3.5 g/24 h) was present in 2 unrelated patients (12%) with serum albumin levels of 26 and 32 g/L (Table 1, family 9 and 16). These patients did not receive immunosuppressive therapy because they had a positive family history of kidney disease, suggesting a genetic cause of the nephrotic syndrome. One index patient (family 11), published previously with familial benign hematuria, showed kidney function deterioration during follow-up in our center.^1^

**Table 1:** Clinical and Genetic Characteristics in 17 Index Patients With 1 Mono-allelic *COL4A3* or *COL4A4* Variant

COL4A3														
Index Patients (Fam)	cDNA^a^	Protein Level	Current Variant Classification∗	Sex	Age at Presentation (y)^c^	eGFR at Presentation^d^	Hematuria at Presentation	Proteinuria at Presentation	Serum Albumin at Presentation (g/L)	Age at End FU (y)	eGFR at End FU^d^	Proteinuria at End FU	Extrarenal Symptoms	Previous Reports /Novel Variant
1	c.2083G>A	p.Gly695Arg	Pathogenic (PS4, PM1, PM2, PM3, PP1, PP3)	M	4	Normal#	Yes	No	Normal	55	59	0.8 g/10 mmol creatinine	none	11, 34, 26-29
2	c.2083G>A	p.Gly695Arg	Pathogenic (PS4, PM1, PM2, PM3, PP1, PP3)	F	37	92	Yes	1.6 g/24 h	32	43	83	4.1 g/10 mmol creatinine	none	11, 34, 26-29
3	c.2083G>A	p.Gly695Arg	Pathogenic (PS4, PM1, PM2, PM3, PP1, PP3)	M	51	103	Yes	Yes	n/a	56	94	2.2 g/24h	none	11, 34, 26-29
4	c.2801G>A	p.Gly934Glu	VUS (PM1, PM2, PP3)	M	51	21	Yes	2 g/24 h	42	55	kidney failure	kidney failure	none	**novel**
5	c.4235G>A	p.Gly1412Asp	Likely pathogenic (PM1, PM2, PM3, PP3)	F	45	136	Yes	2.9 g/24 h	36	50	121	Yes#	none	30

*COL4A4*														
Index Patients (Fam)	cDNA^b^	Protein Level	Current Variant Classification∗	Sex	Age at Presentation (y)^c^	eGFR at Presentation^d^	Hematuria at Presentation	Proteinuria at Presentation	Serum Albumin at Presentation (g/L)	Age at End FU (y)	eGFR at End FU^d^	Proteinuria at End FU	Extrarenal Symptoms	Previous Reports /Novel Variant
6	c.1505dup	p.Gly503fs	Likely pathogenic (PVS1, PM2)	F	47	101	Yes	0.8 g/24 h	38	57	68	0.4 g/10 mmol creatinine	none	**novel**
7	c.1571G>A	p.Gly524Glu	Pathogenic (PS4, PM1, PM2, PM3, PP1, PP3)	F	47	58	Yes	0.2 g/24 h	n/a	60	37	0.6 g/24 h	none	**novel**
8	c.1571G>A	p.Gly524Glu	Pathogenic (PS4, PM1, PM2, PM3, PP1, PP3)	F	40	99	Yes	No	n/a	67	44	1.4 g/24 h	none	**novel**
9	c.2690G>A	p.Gly897Glu	Pathogenic (PS4, PM1, PM2, PM3, PP1, PP3, PP5)	F	42	95	Yes	5.9 g/24 h	26	50	73	5.3 g/10 mmol creatinine	none	1, 30, 31
10	c.2690G>A	p.Gly897Glu	Pathogenic (PS4, PM1, PM2, PM3, PP1, PP3, PP5)	F	19	112	Yes	1.2 g/24 h	n/a	35	96	2.2 g/24 h	none	1, 30, 31
11^e^	c.2690G>A	p.Gly897Glu	Pathogenic (PS4, PM1, PM2, PM3, PP1, PP3, PP5)	M	6		Yes	Yes#	n/a	42	9	4.1 g/24 h	Hearing + vision	1, 30, 31
12	c.2690G>A	p.Gly897Glu	Pathogenic (PS4, PM1, PM2, PM3, PP1, PP3, PP5)	F	27	108	Yes	0.1 g/10 mmol creatinine	42	32	117	0.4 g/10 mmol creatinine	none	1, 30, 31
13	c.2690G>A	p.Gly897Glu	Pathogenic (PS4, PM1, PM2, PM3, PP1, PP3, PP5)	M	43	104	Yes	0.1 g/10 mmol creatinine	n/a	52	88	0.2 g/10 mmol creatinine	none	1, 30, 31
14	c.2690G>A	p.Gly897Glu	Pathogenic (PS4, PM1, PM2, PM3, PP1, PP3, PP5)	M	45	103	Yes	No	n/a	52	84	0.1 g/10 mmol creatinine	none	1, 30, 31
15	c.2690G>A	p.Gly897Glu	Pathogenic (PS4, PM1, PM2, PM3, PP1, PP3, PP5)	M	39	51	Yes	2-3 g/24 h	n/a	59	kidney failure	kidney failure	none	1, 30, 31
16	c.3532G>A	p.Gly1178Ser	VUS (PM1, PM2, PP3)	F	38	106	Yes	3.5 g/24 h	32	47	101	3.7 g/24 h	none	**novel**
17	c.3706G>A	p.Gly1236Arg	VUS (PM1, PM2, PP3)	M	55	49	Yes	0.9 g/24 h	41	58	45	0.9 g/24 h	none	**novel**

Abbreviations: eGFR, estimated glomerular filtration rate; F, female; Fam, family; FU, follow-up; M, male; n/a, not available; VUS, variant of unknown significance.

∗ Pathogenicity of detected variants was assessed using the American College of Medical Genetics and Genomics (2015) guidelines.^24^

# Not further quantified.

a Reference sequence used is NM_000091.4.

b Reference sequence used is NM_000092.4.

c Age at presentation was determined retrospectively based on first reported symptom (glomerular hematuria/proteinuria).

d Calculated using Chronic Kidney Disease Epidemiology Collaboration (CKD-EPI) formula.

e This patient and his family (family 11) were reported previously by Lemmink et al.^1^

**Table 2:** Clinical Findings at Presentation of 17 Index Patients and 25 Family Members With a Mono-allelic DNA Variant in *COL4A3* or *COL4A4*

	Index Patients	Family Members
Total number, n	17	25
Positive family history, n (%)	17 (100%)	—
Median age at presentation^a^, y (range)	43 (4-55)	39 (8-66)
Sex, n (%)		
Female	9 (53%)	16 (64%)
Male	8 (47%)	9 (36%)
Hearing loss, n (%)		
Yes	1 (6%)	1 (4%)
No	16 (94%)	24 (96%)
Ocular findings^b^, n (%)		
Yes	1 (6%)	0 (0%)
No	16 (94%)	25 (100%)
Microscopic hematuria, n (%)		
Yes	17 (100%)	20 (80%)
No	0 (0%)	1 (4%)
Unknown	-	4 (16%)
Proteinuria, n (%)		
No	3 (17.5%)	3 (12%)
30-300 mg /24 h or /10 mmol creatinine	3 (17.5%)	3 (12%)
≥300 mg /24 h or /10 mmol creatinine	11 (65%)	14 (56%)
Unknown	-	5 (20%)
Median age at last FU, y (range)	52 (32 – 67)	54 (13 – 82)
CKD stage at end of FU (eGFR using CKD-EPI), n (%)		
Stage 1 (eGFR >90 mL/min/1.73 m^2^)	5 (29%)	7 (28%)
Stage 2 (eGFR 60-89 mL/min/1.73 m^2^)	5 (29%)	7 (28%)
Stage 3 (eGFR 30-59 mL/min/1.73 m^2^)	4 (24%)	4 (16%)
Stage 4 (eGFR 15-29 mL/min/1.73 m^2^)	0 (0%)	1 (4%)
Stage 5 (eGFR <15 mL/min/1.73 m^2^)	3 (18%)	4 (16%)
Unknown	—	2 (8%)

Abbreviations: CKD-EPI, Chronic Kidney Disease Epidemiology Collaboration; eGFR, estimated Glomerular Filtration Rate; FU, follow-up.

a Age at clinical presentation was determined retrospectively based on first symptom (glomerular hematuria and/or proteinuria) attributable to *COL4A3* or *COL4A4* variant.

b Under dot-and-flecks retinopathy.

**Figure 1:**
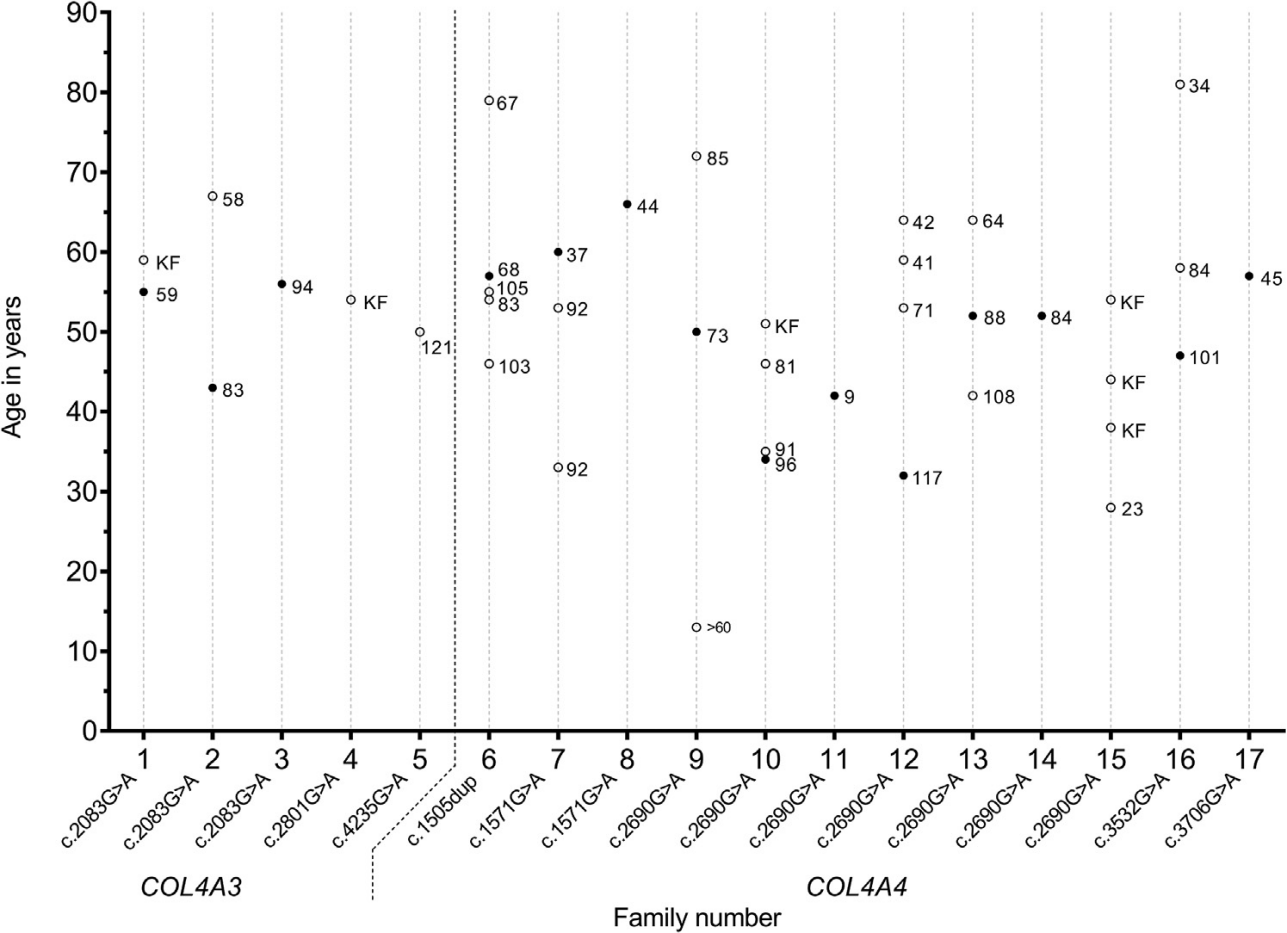
**Estimated glomerular filtration rate (ml/min/1.73** **m**^**2**^**) calculated using the CKD-EPI formula at age of last follow-up for 40 patients with a mono-allelic *COL4A3* or *COL4A4* variant from 17 families.** Each line on the x-axis depicts one family. CKD, chronic kidney disease; CKD-EPI, Chronic Kidney Disease Epidemiology Collaboration; KF, kidney failure (eGFR <15 mL/min/1.73 m^2^ / CKD stage 5); • index patient; o family member.

Segregation analysis was performed in families of 11/17 index patients; in 3 families, informed consent could not be obtained, and in 3 other families, no referral for genetic counseling was made. After inclusion of 25 affected family members, the total study cohort comprised 42 patients with 1 heterozygous variant in *COL4A3* or *COL4A4* (Table 2). At the end of follow-up (median age 54 years, range 13-82 years), 27 patients (64%) had proteinuria, and 16 patients (43%) had an eGFR below 60 mL/min/1.73 m^2^. Six of these 16 patients progressed to kidney failure at a median age of 53 years (range 38-63). A large inter- and intrafamilial difference in eGFR at end of follow-up was observed (Fig 1). Only 1 index patient (family 11) suffered from bilateral high-frequency sensorineural hearing loss and ocular abnormalities (dot-and-flecks retinopathy), characteristic of Alport syndrome. One other patient (sibling of the index patient in family 10) had hearing problems (not further specified) but no known ocular abnormalities and no impairment of kidney function.

### *COL4A3* and *COL4A4* Genotype

Among 17 index patients with a mono-allelic variant in *COL4A3* or *COL4A4*, 16 had a substitution of a glycine amino acid within a collagen triple helix repeat (Gly-X-Y collagenous repeat), and 1 patient had a frameshift variant. In total, 8 different variants were found, 3 in *COL4A3* and 5 in *COL4A4,* of which 5 were novel. Segregation analysis confirmed co-segregation of the variant with the kidney phenotype compatible with an autosomal dominant inheritance pattern in all 11 families available for segregation analysis. Using the American College of Medical Genetics and Genomics guidelines, 3 of the 8 different variants in index patients were classified as pathogenic, 2 as likely pathogenic, and another 3 as variants of unknown significance (Table 1). In family 7 and 8, segregation analysis contributed to the re-classification of the *COL4A4* c.1571G>A (p.Gly524) variant from ‘likely pathogenic’ to ‘pathogenic’. We were not able to establish a genotype-phenotype correlation in the current small series of patients, comparing phenotypes of patients with *COL4A3* variants with *COL4A4* variants and patients with missense variants with non-missense variants (Table 3).

**Table 3:** Genotype-Phenotype Correlations for Affected Gene and Variant Type Among 17 Index Patients and 25 Family Members With a Mono-allelic *COL4A3* or *COL4A4* Variant

	COL4A3	COL4A4	*P*^a^
Number of individuals, n	8	34	
Number of families, n	5	12	
Median age at presentation^b^, y (range)	51 (4-66)	39 (6-61)	0.14
Median eGFR at presentation^c^, mL/min/1.73 m^2^ (range)	86 (14-136)	101 (36-128)	0.32
Proteinuria at presentation, n (%)	7 (100%)	22 (73%)	0.31
Extrarenal findings^d^, n (%)	0 (0%)	2 (6%)	1.00
Median age at last FU, y (range)	55 (43-67)	52 (13-82)	0.38
Median eGFR at last FU^c^, mL/min/1.73 m^2^ (range)	84 (58-121)	73 (9-117)	0.52
End-stage kidney disease, n (%)	2 (25%)	4 (12%)	0.30
Proteinuria at last FU, n (%)	7 (100%)	20 (67%)	0.16

	Missense	Non-missense	*P*^a^
Number of individuals, n	37	5	
Number of families, n	16	1	
Median age at presentation^b^, y (range)	39 (4-66)	48 (38-54)	0.19
Median eGFR at presentation^c^, mL/min/1.73 m^2^ (range)	95 (14-136)	102 (101-116)	0.32
Proteinuria at presentation, n (%)	25 (78%)	4 (80%)	1.00
Extrarenal findings^d^, n (%)	2 (5%)	0 (0%)	1.00
Median age at last FU, y (range)	52 (13-82)	55 (46-80)	0.40
Median eGFR at last FU^c^, mL/min/1.73 m^2^ (range)	73 (9-121)	83 (67-105)	0.30
End-stage kidney disease, n (%)	6 (16%)	0 (0%)	0.57
Proteinuria at last FU, n (%)	24 (75%)	3 (60%)	0.60

*Note:* Percentages were calculated over all cases with available information.

Abbreviations: eGFR, estimated glomerular filtration Rate; FU, follow-up.

a *P* values were calculated using Mann-Whitney *U* test for continuous variables and Fisher exact test for categorical variables.

b Age at clinical presentation was determined retrospectively based on first symptom (glomerular hematuria and/or proteinuria) attributable to *COL4A3* or *COL4A4* variant.

c Calculated using the Chronic Kidney Disease Epidemiology Collaboration (CKD-EPI) formula.

d Auricular or ocular findings.

### Pathological Findings

A kidney biopsy had been performed in 11 index patients and 3 affected family members from 12 families, at ages ranging from 6 to 65 years (Table 4). Reports of evaluation were available for review from all biopsies, and 7 specimens were available for re-evaluation by a kidney pathologist. A classifying histopathological diagnosis was established in 10 out of 14 patients (FSGS [n = 6], thin basement membrane nephropathy [n = 2], Alport syndrome [n = 1], and minimal change nephropathy [n = 1]) (Table 4). No classifying diagnosis could be made in 4 patients: 1 patient with a likely pathogenic variant in *COL4A3* (family 5), 1 patient with a pathogenic variant in *COL4A4* (family 15), and 2 patients with different novel variants of unknown significance in *COL4A4* (family 16 and 17). Among the patients with a variant of unknown significance, 1 had a biopsy classified as FSGS (family 4), and no classifying diagnosis could be established in 2 others (family 16 and 17). Electron microscopy was performed in 11 out of 14 patients at a median age of 44 years (range 6-65 years), revealing abnormalities in all but 1 patient, consisting of segmental thinning of the GBM without lamellation in 7 patients and diffuse GBM thinning in 2 patients (Table 4). Typical lamellation, as seen in Alport syndrome, was observed in only 1 patient, who was diagnosed with hearing loss and ocular lesions (index patient of family 11).

**Table 4:** Pathological Characteristics (Based on Kidney Biopsy Reports or Re-evaluation by Kidney Pathologist and Clinical Characteristics at Time of Kidney Biopsy in 11 Index Patients and 3 Affected Family Members With 1 Mono-allelic *COL4A3* or *COL4A4* Variant

Index Patients												
Clinical Characteristics at Time of Kidney Biopsy					*COL4A3/COL4A4* Variants	Diagnosis Based on Kidney biopsy	Light Microscopy	Immunofluorescence	Electron Microscopy			
Fam	Age (y)	Creatinine (mg/dL)	eGFR (mL/min/1.73 m^2^)	Proteinuria	cDNA		Number of Glomeruli		Podocyte Effacement	GBM Lamellation	GBM Thinning	GBM Thickness, (μm) Median (Range)
2^a^	37	0.70	>90	3.1g/10 mmol creatinine	*COL4A3* c.2083G>A∗	FSGS	28	neg	partial	no	segmental thinning	0.23 (0.14-0.35)
3^a^	54	0.88	89	2 g/24 h	*COL4A3* c.2083G>A∗	FSGS	15	neg	partial	no	segmental thinning	0.31 (0.1-0.55)
4^a^	51	2.6	26	2 g/24 h	*COL4A3* c.2801G>A∗∗∗	FSGS	12	neg	partial	no	segmental thinning	0.28 (0.13-0.42)
5	46	normal#	normal#	2.9 g/24 h	*COL4A3* c.4235G>A∗∗	No classifying diagnosis^b^	15	neg	none	no	segmental thinning	—
8^a^	65	1.10	50	1.4 g/24 h	*COL4A4* c.1571G>A∗∗	FSGS	26	neg	partial	no	segmental thinning	0.42 (0.27-0.65)
9^a^	42	0.77	82	6.5 g/24 h	*COL4A4* c.2690G>A∗	TBMN	30	neg	partial	no	diffuse thinning	0.26 (0.13-0.46)
10	29	normal#	normal#	3 g/24 h	*COL4A4* c.2690G>A∗	MCN	13	neg	complete	no	normal	—
11	6	1.03	normal#	yes#	*COL4A4* c.2690G>A∗	Alport	30	neg	none	yes	—	
15	40	1.64	46	2-3 g/24 h	*COL4A4* c.2690G>A∗	No classifying diagnosis^b^	8	neg	EM not performed			
16^a^	44	0.72	>60	3.5 g/24 h	*COL4A4* c.3532G>A∗∗∗	No classifying diagnosis^b^	4	neg	partial	no	segmental thinning	—
17^a^	56	1.55	47	0.9 g/24 h	*COL4A4* c.3706G>A∗∗∗	No classifying diagnosis^b^	6	neg	partial	no	segmental thinning	—

Family Members												
Fam	Age (y)	Creatinine (mg/dL)	eGFR (mL/min/ 1.73 m^2^)	Proteinuria	cDNA	Diagnosis Based on Kidney Biopsy	Number of Glomeruli	Immunofluorescence	Podocyte Effacement	GBM Lamellation	GBM Thinning	GBM Thickness, (μm) Median (Range)
7	25	0.69	>90	n/a	*COL4A4* c.1571G>A∗	TBMN	n/a	neg	n/a	n/a	diffuse thinning	—
10	43	n/a	n/a	yes#	*COL4A4* c.2690G>A∗	FSGS	8	neg	EM not performed			
15	39	1.75	32	1.3 g/24 h	*COL4A4* c.2690G>A∗	FSGS	14	neg	EM not performed			

Abbreviations: EM, electron microscopy; Fam, family; FSGS, focal segmental glomerulosclerosis; GBM, glomerular basement membrane; MCN, minimal change nephropathy; n/a, not available; neg, negative; TBMN, thin basement membrane nephropathy. Patient 11 was the only patient with an extrarenal phenotype (Table1).

*Note:* Conversion factors for serum creatinine in mg/dL to μmol/L, ×88.4.

∗ Pathogenic variant.

∗∗ Likely pathogenic variant.

∗∗∗ Variant of unknown significance.

# Not further quantified.

a Biopsies available for re-evaluation by kidney pathologist.

b No glomerular lesions.

To investigate whether EM can distinguish patients with a mono-allelic variant in *COL4A3* or *COL4A4* from patients with other glomerular diseases, we revised EM images from the patients with a mono-allelic variant in *COL4A3* or *COL4A4* (n = 7) and compared these with EM images from patients with other forms of FSGS (n = 14; Table 5). The 7 patients with *COL4A3/COL4A4* variants encompassed 4 patients with different pathogenic variants in *COL4A3/COL4A4* and 3 variants of unknown significance in *COL4A3/COL4A4*. The GBM was found to be significantly thinner in patients with a mono-allelic variant in *COL4A3/COL4A4* (median of 0.29 μm (interquartile range 0.22-0.35 μm) compared to patients with idiopathic FSGS and other hereditary causes (median of 0.36 μm [interquartile range 0.29-0.45 μm]) (*P* < 0.01). Kidney biopsies from patients with idiopathic FSGS showed more podocyte foot process effacement compared to biopsies from patients with mono-allelic variants in *COL4A3* or *COL4A4*, which likely reflects the difference in proteinuria between these patient groups (8.6 vs 2.3 g/24 h, respectively). Injury of endothelial cells was present in both (Table 5).

**Table 5:** Revision of Electron Microscopy Images by a Kidney Pathologist of 7 patients With a Mono-allelic *COL4A3* or *COL4A4* Variant and Blind Comparison With 14 Controls

Patient ID	Sex (M/F)	Age at Kidney Biopsy (y)	GBM Thickness (μm) Median (Range)	Total Number Available EM Images	Total Number Points in GBM Measured (n)	GBM Thickness: Consistency	Podocyte Foot Effacement	Endothelial Swelling/Injury	Clinical and Genetic Findings
**Patients with a mono-allelic *COL4A3/COL4A4* variant**									
Index 2	F	38	0.23 (0.14-0.35)	6	25	Variable	+	+	Mono-allelic *COL4A3* variant c.2083G>A (p.Gly695Arg)
Index 3	M	55	0.31 (0.1-0.55)	5	47	Variable	+	+	Mono-allelic *COL4A3* variant c.2083G>A (p.Gly695Arg)
Index 4	M	52	0.28 (0.13-0.42)	6	25	Variable	+	++	Mono-allelic *COL4A3* variant c.2801G>A (p.Gly934Glu)
Index 8	F	65	0.42 (0.27-0.65)	3	20	Variable	+	+	Mono-allelic *COL4A4* variant c.1571G>A (p.Gly524Glu)
Index 9	F	43	0.26 (0.13-0.46)	7	34	Variable	+	+	Mono-allelic *COL4A3* variant c.2690G>A (p.Gly897Glu)
Index 16	F	45	0.22 (0.14-0.34)	4	24	Variable	+	+	Mono-allelic *COL4A4* variant c.3532G>A (p.Gly1178Ser)
Index 17	M	56	0.31 (0.18-0.47)	5	43	Variable	+	+	Mono-allelic *COL4A4* variant c.3706G>A (p.Gly1236Arg)
**Controls**									
1	M	36	0.49 (0.15-0.71)	5	15	Variable	++	-	iFSGS
2	M	59	0.33 (0.2-0.64)	6	16	Variable	++	++	iFSGS
3	M	42	0.34 (0.17-0.77)	6	22	Highly variable	++	++	iFSGS
4	M	85	0.44 (0.3-0.61)	5	24	Variable	++	+	iFSGS
5	M	50	0.36 (0.23-0.47)	5	21	Low variability	++	-	iFSGS
6	M	23	0.48 (0.25-0.91)	7	31	Variable	++	+	iFSGS
7	M	65	0.31 (0.17-0.48)	7	34	Variable	+	+	SRNS
8	M	36	0.35 (0.21-0.66)	5	28	Variable	++	-	SRNS
9	F	43	0.22 (0.13-0.37)	6	39	Variable	+	+	SRNS
10	M	28	0.38 (0.27-0.63)	6	40	Variable	++	+	SRNS
11	M	17	0.37 (0.36-0.8)	1	3	Low variability	+	-	SRNS Mono-allelic *NPHS2* variant c.686G>A (p.Arg229Gln)^a^
12	F	51	0.31 (0.14-0.7)	6	24	Variable	++	+	Compound heterozygous *NPHS2* variants c.686G>A (p.Arg229Gln) and c.862G>A (p.Ala288Thr)
13	F	18	0.63 (0.4-0.79)	3	5	Consistent	++	+	Compound heterozygous *CRB2* variants c.3313C>T (p.Arg1105Cys) and c.3846_3849del (p.Glu1282fs) Mono-allelic *NPHS1* variant c.3549C>A (p.Tyr1183∗)
14	F	2	0.32 (0.23-0.42)	2	10	Low variability	++	+	Compound heterozygous *COQ2* variants c.590G>A (p.Arg197His) and c.683A>G (p.Asn228Ser)

*Note:* Controls included 14 kidney biopsies: patients with a different hereditary nephropathy diagnosed by whole exome sequencing (WES) in our center (n = 3); patients with a steroid resistant nephrotic syndrome (SRNS) (kidney gene panel by WES negative for a disease-causing DNA variants) (n = 5); and patients with idiopathic FSGS (iFSGS) with a complete or partial remission on prednisone (no genetic test performed) (n = 6). Podocyte effacement was classified as none (-), ≤80% (+), or >80% (++). Endothelial injury was classified as none (-), moderate (+), or severe (++).

Abbreviations: EM, electron microscopy; F, female; GBM, glomerular basement membrane; iFSGS, idiopathic focal segmental glomerulosclerosis; M, male; SRNS, steroid resistant nephrotic syndrome.

a Reference sequence used are: *NPHS2* NM_014625.3, *CRB2* NM_173689.6, *NPHS1* NM_004646.3, *COQ2*: NM_015697.7, *COL4A3* NM_000091.4, *COL4A3* NM_000092.4.

## Discussion

Here, we describe in-depth clinical, pathological, and genetic investigations of 42 patients from 17 families with 8 different mono-allelic variants in *COL4A3* or *COL4A4,* identified with a kidney disorders gene panel using whole exome sequencing in a diagnostic approach. Five of these variants had not been reported in the literature previously. A wide range of clinical phenotypes was observed, ranging from a normal eGFR at age 56 years in one patient to kidney failure at age 38 years in another. No clear genotype-phenotype correlation could be observed. In our re-evaluation of kidney biopsy samples, we found that the GBM was significantly thinner in patients with mono-allelic variants in *COL4A3* or *COL4A4* than in patients with other forms of FSGS.

All index patients presented with persistent or recurrent microscopic hematuria, and 82% (14/17) showed accompanying (micro)albuminuria/proteinuria. A positive family history of (micro)hematuria was recorded for all index patients, and the rates of hematuria and proteinuria in family members (95% and 81%, respectively) were comparable to those in the index patients.^8^ In an excellent systemic review of 777 previously reported patients with mono-allelic *COL4A3/COL4A4* variants by Matthaiou et al,^32^ hematuria was the predominant finding in 95% of patients, whereas proteinuria (defined as >500 mg/day) was documented in 46%. Similar numbers were found by Furlano et al,^22^ who reported hematuria in 92% and proteinuria in 65%. The rate and age of onset of kidney failure (14%, median age 53 years), were also in line with previous studies.^22^^,^^32^ We observed sensorineural hearing loss and ocular abnormalities in only 1 of our 42 patients, consistent with the relatively low prevalence of hearing loss (8%-16%) and ocular lesions (1%-3%) described in the literature.^22^^,^^32^

In our series of 17 index patients, we identified 8 different mono-allelic *COL4A3/COL4A4* variants, of which 5 were novel. Three were classified as pathogenic, 2 as likely pathogenic, and 3 as variants of unknown significance. Familial segregation of the DNA variant with glomerular disease was observed in all 11 families investigated. The segregation pattern was consistent with an autosomal dominant mode of inheritance in 4 families. Based on segregation analysis in 2 families, 1 variant (*COL4A4* c.1571G>A) was reclassified from ‘likely pathogenic’ to ‘pathogenic.’ The commonly occurring variant c.2690G>A in *COL4A4* (present in 7 out of 17 families) suggests a founder effect.

Among the 8 different *COL4A3/COL4A4* variants, 7 are documented as missense variants and 1 as a frameshift variant. Remarkably, all missense variants comprised glycine substitutions (Gly to Arg, Asp, Glu, or Ser). Glycine is found at each third residue in the collagen sequence and is critical for the type IV collagen triple helix formation, which is essential for the structure and physiological function of the GBM.^33^ Glycine substitutions, presumed to destabilize the collagen triple helix, are the most common change in X-linked Alport syndrome caused by *COL4A5* variants, but have been found less frequently in autosomal recessive Alport syndrome caused by bi-allelic *COL4A3* or *COL4A4* variants.^34^, ^35^, ^36^, ^37^ Interestingly, glycine substitutions with arginine, aspartic acid, glutamic acid, tryptophan, or valine have been shown to result in more severe disease compared to substitutions with alanine, serine, or cysteine.^38^^,^^39^ Other studies have also demonstrated that non-missense variants in X-linked and autosomal recessive Alport syndrome are associated with more severe disease and earlier age of kidney failure compared to missense variants.^34^^,^^36^^,^^37^^,^^40^ In our study, we were not able to identify genotype-phenotype correlations based on the type of DNA variant or the gene involved. However, our cohort was relatively small, and the power to detect correlations with small effect sizes was limited. In the patient with typical extrarenal features and GBM lamellation (index of family 11), whole exome sequencing using a kidney disorders gene panel identified *COL4A4* variant c.2690G>A (p.Gly897Glu), but no additional variants in *COL4A3/COL4A4/COL4A5* or other podocyte-related genes. At present, DNA variants of individuals are not sufficient for an accurate prediction of their phenotypic outcome.

The most common histopathological diagnosis in our cohort was FSGS, which was diagnosed in 6 out of 10 patients with a classifying histopathological diagnosis. EM revealed segmental or diffuse thinning of the GBM as an isolated finding in 7 out of 11 patients (64%) and GBM lamellation, classical for Alport syndrome, in only 1 patient. Comparable pathologic findings were reported in previous studies, with diffuse GBM thinning in 81% (141/174) of patients with mono-allelic variants in *COL4A3/COL4A4*, an isolated finding in 54% of patients and accompanied by other findings in 27%, and lamination of the GBM in 7% (12/174) of patients.^22^^,^^32^ In line with the study by Furlano et al,^22^ we showed that the GBM is statistically significantly thinner in patients with *COL4A3/COL4A4* variants (<0.30 μm) than in patients with idiopathic FSGS.

The present study describes a selected cohort of patients in whom hematuria was accompanied by proteinuria and a positive family history, which inevitably limits the generalizability of our results. Furthermore, the relatively small size of our cohort limited our power to detect genotype-phenotype correlations. However, this study benefits from a thorough clinical, genetic, and histopathological evaluation, which allowed us to investigate correlations between genotype and both clinical and histopathological features. Even using our small number of available kidney biopsies, we were able to identify a significant difference in GBM thickness between patients with *COL4A3/COL4A4* variants and other causes of FSGS, which is a finding that could be used in diagnostic workup.

The wide spectrum of clinical and histopathological findings of our patients with a mono-allelic variant *COL4A3* or *COL4A4* further demonstrates the challenge of finding uniform and univocal nomenclature for patients with mono-allelic variants in these genes.^41^^,^^42^ At present, we support the previously proposed term ‘type IV collagen (*COL4A3/COL4A4*) nephropathy’ as a broad and overlying diagnosis for all patients with at least glomerular hematuria and a mono-allelic variant *COL4A3* or *COL4A4*.^43^ In the future, an accurate molecular diagnosis of kidney disorders related to *COL4A3/COL4A4* variants and accompanying modifying genes might further contribute to the classification of these kidney diseases and will have implications for monitoring, treatment, and genetic counseling of patients and at-risk family members.

In conclusion, this study confirms that mono-allelic *COL4A3* or *COL4A4* variants are associated with a wide clinical and pathological spectrum of kidney disease, ranging from isolated microscopic hematuria to kidney failure. Genotype-phenotype correlations could not be established in this study, and accurate prediction of a patient’s phenotype based on the identified DNA variant is not yet possible. The rapid development and application of next generation sequencing facilitates early and increased molecular diagnoses of mono-allelic variants in *COL4A3/COL4A4* in the general population. Long-term follow-up of affected patients and family members with these variants will facilitate larger genotype-phenotype studies and may lead to elucidation of the pathogenic mechanisms underlying the extreme phenotypic heterogeneity. The identification of genetic modifiers and biomarkers involved in the recognition and detection of kidney disease progression are crucial for developing preventative or curative therapies.
